# Combined Optimized Effect of a Highly Self-Organized Nanosubstrate and an Electric Field on Osteoblast Bone Cells Activity

**DOI:** 10.1155/2019/7574635

**Published:** 2019-03-21

**Authors:** Diana V. Portan, Despina D. Deligianni, George C. Papanicolaou, Vassilis Kostopoulos, Georgios C. Psarras, Minos Tyllianakis

**Affiliations:** ^1^Department of Mechanical and Aeronautics Engineering, Composite Materials Group, University of Patras, Patras 265 00, Greece; ^2^Department of Mechanical and Aeronautics Engineering, Laboratory of Biomechanics and Biomedical Engineering, University of Patras, Patras 265 00, Greece; ^3^Department of Materials Science, University of Patras, Patras 265 00, Greece; ^4^Department of Shoulder and Elbow Surgery, University Hospital of Patras, Rio, Greece

## Abstract

The effect of an electric field within specific intensity limits on the activity of human cells has been previously investigated. However, there are a considerable number of factors that influence the in vitro development of cell populations. In biocompatibility studies, the nature of the substrate and its topography are decisive in osteoblasts bone cells development. Further on, electrical field stimulation may activate biochemical paths that contribute to a faster, more effective self-adjustment and proliferation of specific cell types on various nanosubstrates. Within the present research, an electrical stimulation device has been manufactured and optimum values of parameters that led to enhanced osteoblasts activity, with respect to the alkaline phosphatase and total protein levels, have been found. Homogeneous electric field distribution induced by a highly organized titanium dioxide nanotubes substrate had an optimum effect on cell response. Specific substrate topography in combination with appropriate electrical stimulation enhanced osteoblasts bone cells capacity to self-adjust the levels of their specific biomarkers. The findings are of importance in the future design and development of new advanced orthopaedic materials for hard tissue replacement.

## 1. Introduction

In a physiologic condition, all human cells perform in a low frequency electric environment. Although the influence of electromagnetic fields on biological systems has attracted attention in recent years, few publications describe the effects of interaction and the underlying mechanisms involved. Initially, focus was given to the genotoxic effect of electric and magnetic fields [1]. However, about the same period it was also found that under certain conditions there was no effect of electric and/or magnetic field on DNA structure and function in cultured human cells. Exposure of cultured cells to 50 Hz electric (0.2-20 kV/m), magnetic (0.002-2 G), or combined electric and magnetic fields for up to 24 h did not result in the production of detectable DNA lesions. The rate of cell growth was also unaffected as well as the intracellular ATP and NAD^+^ levels. These results proved that, under an intensity threshold, magnetic and electromagnetic fields are not geno- and cytotoxic in cultured mammalian cells [2]. Moreover, a more recent study has shown that the external electric field can positively influence the membrane electrical activity and perhaps the insulin secretion of pancreatic *β*-cell when its amplitude exceeds a threshold value. Furthermore, it has been shown that different waveforms have distinct effects on the *β*-cell membrane electrical activity and the characteristic features of the excitation like frequency would change the interaction mechanism [3]. Generally, focus was given to identifying the intensity and field parameters that result in a positive effect on human cells. Between different cell types, bone cells were several times chosen, since they may be collected from patients that are submitted to bone surgery through a simple isolation procedure [4]. Experiments for the determination of the electrical parameters that lead to optimal expression of several bone-related genes in cultured human bone indicated that a capacitively coupled electric field of 60 kHz, 20 mV/cm, 50% duty cycle for 2 hours duration per day significantly upregulated mRNA expression of several bone morphogenetic proteins as well as fibroblast growth factor, osteocalcin, and alkaline phosphatase. The clinical relevance of these findings in the context of a noninvasive treatment modality for delayed union and nonunion fracture healing was seriously taken into consideration.

In practice, clinical applications of electric fields for bone repair have been reported since the 1980s. An evaluation of the use of constant direct current in treating acquired nonunion of bones was expanded to 12 participants. In this study, 83.7 percent nonunion cases achieved solid bone union when treated with adequate electricity. Also, complications of the electrical treatment were minor and there was no deep infection resulting from this procedure in patients. It was concluded that given proper electrical parameters and proper cast immobilization, a rate of bone union comparable to that seen with bone-graft surgery was achieved [5]. The practicing orthopaedic surgeon utilizing constant direct current to treat nonunion should, by proper fracture management and following the biophysical principles described herein, achieve a rate of union comparable to that of bone-graft surgery, with a lower associated risk [6].

In situations where an implant is mandatory, the potential of bone tissue regeneration may be amplified by introducing electrically conductive biocompatible implant surfaces. In vitro, the observation of different bone cell populations seeded on implant materials can offer significant information on the effects of substrate characteristics on their bioactivity. An appropriate electric field may improve the proliferation and differentiation of osteoblasts and the rehabilitation of injured bones. In the treatment of various bone injuries, electrical stimuli promote callusogenesis and play an important role in the regulation of the adhesion, proliferation and differentiation of osteoblasts. Moreover, a combination of conductive substrates and field stimulation may lead to an ideal osteogenic differentiation rate and enhanced activity of these types of bone cells [7, 8]. It has been previously demonstrated that an electroactive substrate that mediated electrical stimulus determined the differentiation of preosteoblast bone cells into mature-osteoblasts [9]. Within this context, the purpose of the present research was to observe the combined effect of electrical stimulation and various substrates on osteoblast bone cells activity. Moreover, the aim was to define the electrical threshold, as well as the nature and the type of substrate topography that determine an improved cell behaviour.

## 2. Materials and Methods

### 2.1. Titanium Substrates

**Figure 1:**
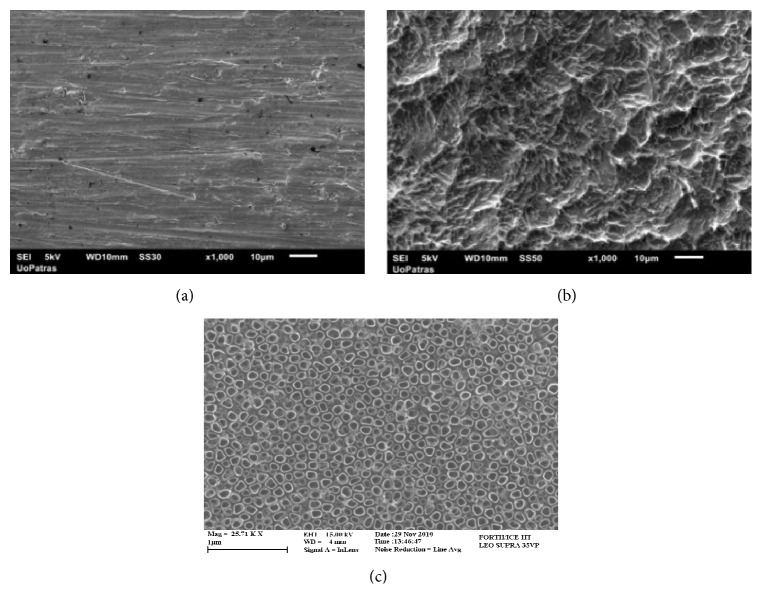
The three types of titanium substrates involved in the study: (a) smooth, unprocessed titanium; (b) rough, anodized titanium; and (c) anodic titanium dioxide nanotubes.

Four substrate categories have been used: (1) tissue culture polystyrene (TCP) as a control material; (2) pure titanium plate as provided by the supplier (Alfa Aesar S. A. Massachusetts) (Figure 1(a)); (3) pure titanium plate with increased surface roughness (Figure 1(b)); and (4) titanium dioxide nanotubes (TNTs) layer synthesized on a pure titanium plate (Figure 1(c)). The increased roughness and the titanium dioxide nanotubes topographies have been obtained by electrochemical anodization, as described in [10–13].

### 2.2. Electrical Measurements

Electrical properties of the employed substrates were determined according to the American Society for Testing Materials standards (ASTM D150), by means of Alpha N-Frequency Response Analyzer (Novocontrol Technologies) operating in the frequency range 10^−1^ to 10^6^ Hz, at ambient temperature. The amplitude of the applied voltage was 1 V and specimens were placed between two gold coated electrodes in a parallel plate capacitor configuration in the BDS 1200 cell (Novocontrol Technologies).

### 2.3. Isolation of Osteoblast Bone Cells

Human bone cells (osteoblasts) were isolated from residual bone obtained from a 47 years old female patient, during knee surgery. The cell suspension was cultured until confluence in medium described in [14, 15]. During multiplication, proliferation, and while seeded on TCP substrate, osteoblasts cultures were observed with an inverted optical microscope (Nikon Diaphot).

### 2.4. Stimulation Device and Parameters

**Figure 2:**
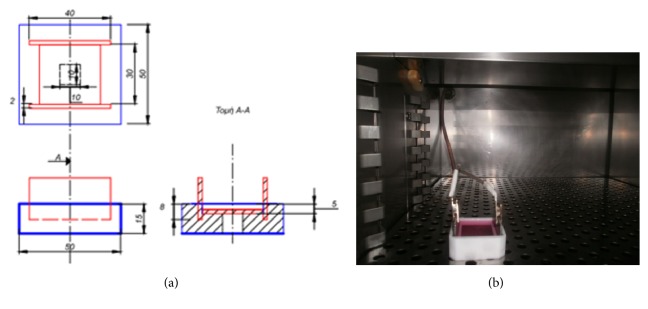
Electricity stimulation: (a) computer design and (b) setup in the incubator.

An electricity stimulation device has been designed (Figure 2(a)) and manufactured using Teflon and titanium materials; these materials were chosen since they are nontoxic when in contact with human cells and tissues. Furthermore, they may be easy sterilized under both pressure and high temperatures in an autoclave. Moreover, titanium is electrically conductive. The two vertical titanium electrodes coming in contact with the titanium bottom plate mediated electrons transfer to the culture medium (Figure 2(b)). Substrates were also in contact with the titanium bottom and were covered by medium so that cell populations may be maintained for several hours in the device, under electrical stimulation. The stimulation device was installed in the incubator as observed in Figure 2(b) and allowed to electrically stimulate the human bone cells through both the substrate and the medium.

The human cell populations seeded on the different substrates were exposed to an electric field with potential between 0.3 and 1 V for periods of 20 minutes to maximum 2 hours, as described in the section of Results and Discussion. Prior to exposure, osteoblasts were seeded on the substrates and kept in the incubator for one night. Alkaline phosphatase and total protein levels were further measured after one and three days of incubation.

### 2.5. Cell Response

The examination of cells morphology on TCP was performed by optical microscopy. The cell response was evaluated by quantification of alkaline phosphatase (ALP) and total protein (TP) in the cell populations after exposure to an optimum electric treatment selected after performing several experiments and analysing cells morphology. ALP activity is the most widely recognized biochemical marker for early osteoblast differentiation. ALP enzyme activity (Sigma Aldrich, P7998) was assessed after 1 and 3 days of cell culture. Further on, the TP is an indication of cells proliferation. The intracellular quantity for TP synthesis was assessed after 1 and 3 days of cell culture by Total Protein Kit (Sigma TP0400). An Infinite F200PRO UV/visible Spectrometer has been used to detect the ALP and the TP levels at 405 and 600 nm wavelengths, respectively. Experimental data were collected from three separate experiments and were expressed as mean values 6SD. They were analysed using SPSS 14.0 software (SPSS, USA). The level of significance was calculated using Student's test for single comparisons and ANOVA for multiple comparisons. Statistical significance was assumed at the 95% confidence limit or greater (p<0.05).

## 3. Results and Discussion

### 3.1. Electrical Properties of the Substrates

The presence of anodized titanium and TNTs layer on the surface of pure titanium plates transforms the substrate from conductive to dielectric. The resistivity of the anodized titanium substrate, at the lowest measured frequency of 0.1 Hz, was found to be 2.92 × 10^9^ Ohm·cm, while the relative value of the TNTs substrate was 2.46 × 10^11^ Ohm·cm. The increase by two orders of magnitude of resistivity in the case of the TNTs indicates an enforcement of the insulating properties of the substrate. Moreover, the configuration of layers corresponds to capacitors connected in series carrying the same amount of electric charge in absolute value. In this case, the ratio of the electric field in the layers is inverse proportional to the ratio of the corresponding dielectric permittivity (or dielectric constant) of each layer, according to

(1) $$\begin{array}{c} \frac{E_{anod}}{E_{TNT}}=\frac{\varepsilon_{TNT}}{\varepsilon_{anod}} \end{array}$$

where* E*_*anod*_,* E*_*TNT*_ and* ε*_*anod*_,* ε*_*TNT*_ are the electric field and dielectric constant, respectively. The measured values of* ε*_*anod*_ and* ε*_*TNT*_, at the lowest applied frequency, were 3.82 and 160.00 being in accordance with the enhanced of the insulating behaviour of the TNTs substrate. Following (1),* E*_*TNT*_ is proportional to the* ε*_*anod*_/*ε*_*TNT*_ ratio which takes the value of 0.024, indicating a significant reduction of the field's intensity.

Further on, biocompatibility tests involving human osteoblasts bone cells use as a standard substrate the tissue culture polystyrene (TCP). Within the present research, osteoblasts populations were seeded on TCP and the adjustment of the electric stimulation parameters (time and voltage) was made after observing osteoblasts morphology with an optical microscope. Several electric protocols were applied. Results are shown in the following section.

### 3.2. Cells Morphology

**Figure 3:**
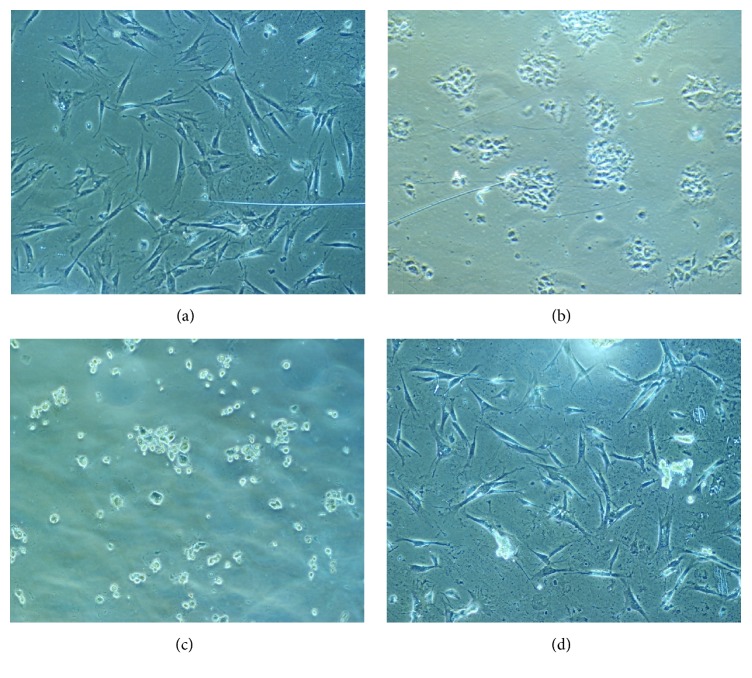
Optical micrographs of osteoblasts on TCP substrate: (a) control sample, no electrical stimulation; (b) electrical stimulation, 1V, 20 min; (c) electrical stimulation, 0.7V, 2 hours; and (d) electrical stimulation, 0.3V, 3 hours.

Figure 3(a) shows the morphology of osteoblast cells seeded on TCP in normal conditions (no electrical stimulation) after one day of incubation. Osteoblasts have a physiologic morphology. Further on, in Experiment 1, cells were subjected to a potential of 1V for 20 minutes. As observed in Figure 3(b) osteoblasts were grouped and their morphology was unusual, indicating that the electrical parameters stopped their development and proliferation and led to cells death. Almost similar results (Figure 3(c)) were found when a lower potential of 0.7V has been applied for two hours. Finally, 0.3V for 3 hours was the ideal potential-time combination that conserved the physiologic morphology of osteoblasts population on the TCP substrates (Figure 3(d)). This parameter combination has been further applied for the stimulation of osteoblasts populations while they were seeded on the three types of titanium substrates: (i) smooth titanium, (ii) rough titanium, and (iii) titanium dioxide nanotubes.

### 3.3. Alkaline Phosphatase Activity and Total Protein Level

**Figure 4:**
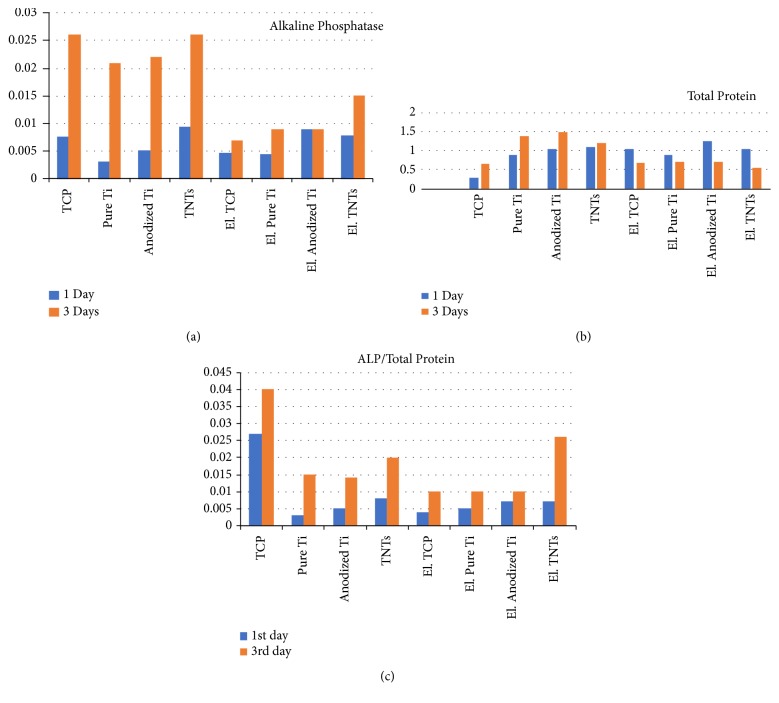
The level of (a) alkaline phosphatase; (b) total protein, and (c) ALP/total protein ratio on the different types of substrates, in nonstimulated and electrically stimulated osteoblasts.

The ALP activity and TP levels have been measured after one and three days of incubation in nonstimulated and electrically stimulated osteoblasts. As previously described in [14, 15], ALP is related to osteoblasts early differentiation, while TP is related to the proliferation and the degree of adhesion of the cells on the substrate. Both parameters are crucial in the development of new biomaterials aimed to induce rapid and efficient osteointegration. Moreover, the two parameters are influenced by several factors such as substrate nature (metallic, ceramic, and plastic), scale (micro or nano), topography (shape; organization), roughness, etc. Various studies are performed to identify the ideal combination of these factors for specific implant applications. In biocompatibility studies of new developed materials, a high “ALP/TP” ratio corresponds to differentiation, a good proliferation, and an appropriate adhesion, assuring maximum cooperation between the living cells and the underlying substrate. The diagrams in Figures 4(a) and 4(b) show the values of the two biomarkers for all substrates, without electrical stimulation of the osteoblasts (TCP, pure titanium, anodized titanium, and TNTs) and with an optimum electrical treatment of 0.3V, for 3 hours (El. TCP, El. Pure Titanium, El. Anodized Titanium, and El. TNTs), after one and three days of incubation.

### 3.4. Alkaline Phosphatase Activity

As observed in the ALP diagram in Figure 4(a), in the nonstimulated cells ALP values are considerably higher after three days, comparing to one day of incubation, which is expected as cells accommodate with the substrate and differentiate in an extended time period. Also, it was previously reported and it is confirmed in the present study that a positive change in osteoblasts behaviour on TNTs resulting in substantially enhanced upregulation of ALP activity exists, suggesting a bone-forming ability at a level as high as TCP [16]. Further on, in electrically stimulated osteoblasts, ALP levels are low for TCP, while they lightly increase in almost all cases when cells are seeded on metallic substrates. A first significant observation is that electricity influences human osteoblasts differentiation when these are seeded on semiconductive substrates. Most common orthopaedic materials are metals; besides other outstanding properties, one of their advantages is that they are electrically conductive. However, conductive polymeric materials are being developed as well for the stimulation of nerve regeneration [17]. Other types of progress in this direction focus on carbon nanotubes (CNTs) based biomaterials [18]. For the moment, these polymeric composites may only be used as coatings in orthopaedic implantology because of their poor mechanical properties, while the most important component remains the metallic one. The ALP levels in osteoblasts populations used in the present investigation confirm that titanium substrates are effective for in vitro bone cells growth in the presence of an electric field, ensuring increased differentiation rate comparing to other substrates.

In the ALP diagram in Figure 4(a), it may be further seen that after three days of incubation in the presence of an electric field, TNTs induce differentiation in osteoblasts at a level much higher than TCP. Moreover, in electrically stimulated osteoblasts the increase in the ALP level from the first to the third day of incubation is not significant comparing to the great difference that may be observed in the case of nonstimulated cells. A second highlight in this investigation is that electricity triggers cells and enables processes that usually need an extended time period, within minutes. This is valid for any type of substrate, considering that the effect may be observed on titanium, as well as on TCP. Finally, the TNTs substrate and the electricity bring maximum efficiency in the system. The ALP level after three days of incubation is much higher in electrically stimulated osteoblasts on TNTs comparing to the one in osteoblasts seeded on other substrates, considerably overpassing even the ALP level in electrically stimulated osteoblasts on TCP control material.

### 3.5. Total Protein Level

The TP level in osteoblasts is normally high when they are seeded on titanium since proteins are involved in cells adhesion to the substrate; metallic materials, due to their nature, induce strong cells adherence. In the case of titanium, Kabaso et al. explained the mechanism through a strong local adhesion due to electrostatic forces that locally trap the osteoblast membrane and hinder the further spreading of osteointegration boundary. Finally, they suggested that the synergy between adhesion and spreading is responsible for successful osteointegration [19]. However, it was commonly accepted that this synergy was guided by substrate characteristics, which triggered cells and determined a specific response. Although human cells do have great capacity to adapt, their biochemical mechanisms are much too complex to be fully understood; for this reason, focus was often made to tailoring substrate parameters to make it biomimetic in order to induce a specific in vitro cells response. The possibility of changing the physiologic parameters of human cells to make them selective and adaptive to diverse materials was considered less feasible.

In the TP diagram in Figure 4(b) it may be observed that, in the absence of an electric field, the TP level is high when osteoblasts are seeded on smooth and rough titanium, as well as on TNTs, while it is very low on TCP. Further on, in the absence of an electric field, the TP level increases after three days of incubation. A high protein level will lead to a low “ALP/TP” ratio and indicates strong anchorage of osteoblasts to the substrate, which limits their freedom of development. On the opposite, electrically stimulated osteoblasts present completely reversed behaviour. The released TP level seems to be adjusted depending on substrate nature, indicating that cells scan the surface and balance their biochemical activity in cooperation with the environment. Thus, after three days of incubation in the presence of the electric field, values of TP in osteoblasts decrease for all substrates. This could be made through a regulating mechanism where adhesion proteins are involved with the purpose to obtain connection with the substrate but, at the same time, to assure the necessary mobility to allow migration. Integrins are the major cell surface receptors for extracellular matrix molecules, which play critical roles in a variety of biological processes. Focal adhesion kinase has long time ago been established as a key component of the signal transduction pathways triggered by integrins [20] and may be the main component of osteoblasts that is affected by the presence of the electric field, thus playing the role of detector in the adjustment mechanism of protein level. On TCP, the TP level is higher in stimulated comparing to nonstimulated cells. Considering that TCP, being a plastic, does not favour osteoblasts adhesion comparing to metals, it may be deduced that cells adjust TP to improve connection and cooperation with this type of substrate. On the other hand, electrically stimulated osteoblasts present decreased TP level when on metallic substrates, thus adjusting adhesion to confer mobility. In the case of TNTs (Figure 4(b)), a phenomenon takes place that has already been described and modelled [15] through the concept of the adhesion efficiency. On metallic materials, a low TP level is preferred, because this will increase the “ALP/TP” ratio. In the present research, a combination of an optimum electric stimulation together with a uniform, highly organized topography of the substrate (TNTs) and for a period of three days of incubation seems to be the ideal parameter merge that positively influence osteoblasts behaviour. While electricity does not substantially enhance osteoblasts activity when these are seeded on TCP, smooth titanium, and rough titanium, it has a great effect on the cells' response on TNTs, and this is reflected in the high value of “ALP/TP” ration observed in Figure 4(c). It has been previously stated that the nanolevel brash like structure and the highly organized topography of the TNTs show impressive light to electricity conversion efficiency in the dye-sensitized solar cells [21]. TNTs with vertically aligned array structures show substantial advantages in solar cells as an electron transport material that offers a large surface area where charges travel linearly along the nanotubes [22]. The same theory may be applied to explain the formation of a homogeneous excitation field under the osteoblasts, closely mimicking the in vivo environment and resulting in increased cells activity and self-adjustment capacity of the human cells.

## 4. Conclusions

The present investigation aimed to find an appropriate combination of parameters related to substrate characteristics (nature and topography) and an optimum electrical stimulation that leads to the proliferation and enhanced activity of osteoblast bone cells. With this purpose, a cell carrying device has been designed and used for the electrical stimulation of the human osteoblast cells that were isolated from osseous tissue obtained during orthopaedic surgery. The osteoblasts were further seeded on four types of substrates: tissue culture polystyrene (TCP) as a control material, smooth titanium, rough titanium, and titanium dioxide nanotubes (TNTs) for one to three days of incubation. A direct current field was applied in the culture medium and a series of protocols for the electrical stimulation of the osteoblasts have been tested to find the appropriate combination of parameters (voltage, time, and substrate) which enhance their activity. It was found that a combined effect of both applied electrical field (0.3V, 3 hours) and specific substrate nature and topography (titanium dioxide nanotubes) leads to increased “alkaline phosphatase/total protein” ratio, which indicates a good cooperation between the human cells and the substrate, as well as appropriate in vitro conditions. It was concluded that electricity triggers osteoblasts and awakens a regulating mechanism that determines the self-adjustment of protein level. The present research shows that osteoblast adaptability function to substrates may be considerably enhanced by applying in their medium an electric field within specific intensity limits. This adaptability mechanism determines an increased cells capacity to scan the underneath material and balance adhesion to get good connection with the substrate, but at the same time enough mobility to allow cells migration. The involved biochemical paths worth being studies in future investigations and are most probably related to processes where adhesion proteins play a key role. Between the studied substrates, titanium dioxide nanotubes, due to their nature and highly organized architecture, contribute to the uniform distribution of the electric field in the medium and under the bone cell population. Three main general conclusions that open future research avenues were highlighted:

- Electricity influences human osteoblasts differentiation, especially when they are seeded on semiconductive substrates. Functionalized advanced biomaterials in orthopaedics should combine materials with good mechanical properties (e.g., metals), that may be manufactured to mimic human bone tissues (e.g., polymers) and that are highly conductive (e.g., carbon-based materials).
- Appropriate electricity stimulation triggers osteoblast and enhances their capacity to adapt to substrates of diverse nature. The regulating mechanism should be studied as a function of involved adhesion proteins.
- The nanolevel brash like structure and the highly organized topography of the TNTs combined with an optimum electrical stimulation lead to the best “alkaline/phosphatase” ratio level in osteoblasts comparing to other substrates (TCP, smooth titanium, and rough titanium). Although the value of this ratio does not overpass the one in cells on TCP in the absence of an electric field, the mechanism worth being further investigated due to its possible implication in human cells biochemical signalling and relation with the surrounding environment.

Finally, the effect of an adjusted intensity of the electric field and the appropriate topography of the substrate on osteoblasts behaviour demonstrate the importance of these two in vitro parameters for the activity of bone-forming cells and may be considered crucial in the manufacturing of advanced biomaterials for orthopaedic applications.

## Data Availability

The SEM micrographs and Excel files that contain data used to support the findings of this study are available from the corresponding author upon request.
